# The Severity of Dependence Scale (SDS) for Codeine: Preliminary Investigation of the Psychometric Properties of the SDS in an Online Sample of Codeine Users From the UK

**DOI:** 10.3389/fpsyt.2021.595706

**Published:** 2021-04-01

**Authors:** Paolo Deluca, Michelle Foley, Jacklyn Dunne, Andreas Kimergård

**Affiliations:** ^1^National Addiction Centre, King's College London, Institute of Psychiatry, Psychology and Neuroscience, London, United Kingdom; ^2^School of Health Sciences, Waterford Institute of Technology, Waterford, Ireland

**Keywords:** psychometric validation, Severity of Dependence Scale, opioid misuse, codeine, addiction

## Abstract

**Objective:** Investigate the psychometric properties of the Severity of Dependence Scale (SDS) for codeine and its association with aberrant codeine related behaviors.

**Design:** A voluntary and uncompensated cross-sectional online survey.

**Setting:** Online population (≥18 years).

**Respondents:** Two hundred and eighty-six respondents (66% women) who had used codeine containing medicines in the last 3 months and were living in the UK.

**Results:** Of the respondents (mean age = 35.4 years, SD = 12.5), more than half were employed. Only 3.5% respondents reported no income. The majority of respondents (45.1%) primarily obtained prescription-only codeine from a consultation with a health professional, whilst 40.9% mainly purchased “over-the-counter” codeine containing medicines in a pharmacy without a medical prescription. Principal component analysis indicated a single factor solution accounting for 75% of the variance. Factor loadings ranged from 0.83 to 0.89. Cronbach's Alpha was high (α = 0.92). Several behaviors relating to codeine use were found to significantly predict probable codeine dependence. These included: daily codeine use in the last 3 months (OR = 66.89, 95% CI = 15.8–283.18); tolerance to codeine (OR = 32.14, 95% CI = 13.82–74.75); problems with role responsibility due to intoxication (OR = 9.89, 95% CI = 4.95–19.78); having sought advice on the internet to manage codeine use (OR = 9.56, 95% CI = 4.5–20.31); history of alcohol or drug treatment (OR = 3.73, 95% CI = 1.88–7.43).

**Conclusions:** The SDS was acceptable and feasible to use to assess probable psychological codeine dependence in an online sample of people using codeine containing medicines. SDS scores were associated with behaviors known to be indicators of codeine dependence. Studies are needed in well-defined populations of people who use codeine to test the different aspects of psychometry of the scale compared against “gold standard” criterion [a diagnosis according to the Diagnostic and Statistical Manual of Mental Disorders (DSM-5)].

## Strenghts and Limitations of This Study

- Advances the understanding of the use of screening tools and scales to assess dependence on codeine containing medicines for research purposes.
- The study recruited a broad cross-section of codeine users in the UK, providing an initial investigation of the psychometric properties of the Severity of Dependence Scale for codeine.
- Online purposive samples have unknown population characteristics which must be recognized when interpreting the findings of the present study.
- Studies in well-defined populations of people using codeine are needed to test different aspects of psychometry of the scale compared against independent “gold standard” criterion.

## Introduction

In the UK, the use of codeine containing medicines and the resulting possibility of dependence and severe health outcomes (1) pose a burden on primary and secondary care, specialized addiction treatment (2) and mortality (3). Codeine is used in form of codeine-based Prescription-Only Medicines (POM) or Pharmacy medicines (P), which contain a lower amount of codeine and may be sold under the supervision of a pharmacist without a medical prescription (sold “over-the-counter”) (4). Codeine is currently controlled under the Misuse of Drugs Regulations 2001 classified as Schedule 5 (Controlled drugs excepted from the prohibition on importation, exportation and possession) (5).

Many codeine containing medicines include a combination of codeine and a non-opioid analgesic such as ibuprofen or paracetamol (6). In 2014, the UK accounted for nearly one-sixth of the global consumption of codeine (7). Sales of codeine containing “over-the-counter” products in packs of 32 tablets more than doubled in the period of 2006 to 2008 from 5.3 to 11.1 mn (8).

During 2007 to 2016, the number of registered drug-related deaths involving codeine increased from 60 to 131 in England and Wales (9). In Scotland, codeine or a codeine-containing compound was implicated in an average of 19 deaths per year between 2003 and 2007, 27 deaths per year between 2008 and 2012, and 33 deaths per year between 2011 and 2015 (10). The accessibility to codeine is under scrutiny in many countries, including the UK, due to concerns of dependence and severe harm from excessive use and overdose of accompanying paracetamol and ibuprofen (11–13). The recent indicators of an emerging “codeine problem” in the UK expose the need for reliable and accurate instruments to identify and treat early signs of codeine dependence to reduce long-term use, mortality, and the economic burden of addiction treatment.

The Severity of Dependence Scale (SDS) is a simple and practical 5-item, 15-point scale used to assess the degree of psychological dependence across several substance classes (14). In research to date, the psychometric properties of the SDS have been investigated in populations using illicit drugs (14–16), alcohol (17), and nicotine (18). Optimal cut-off scores on the SDS for probable psychological dependence, when measured against the presence of a diagnosis obtained from the Diagnostic and Statistical Manual of Mental Disorders (DSM-5), have been determined for amphetamine (19), cocaine (20, 21), benzodiazepines (22), alcohol (23), and cannabis (24). The scale has previously been used to determine the level of probable codeine dependence amongst adults in Australia reporting use of “over-the-counter” codeine (25). Further investigation of the psychometric properties of the SDS for codeine would add understanding and value to the use of the scale for research purposes and possibly in clinical settings.

Using data collected from a cross-sectional, self-completed, online survey of adults who used codeine, the article presents a preliminary investigation of (i) the psychometric properties of the SDS for people living in the UK and (ii) the relation between the scale and behaviors known to be indicators of codeine dependence. Scales to identify people who are codeine dependent which are reliable and simple to administer are currently needed to promote public health.

## Methods

### Ethics

The study received ethics approval granted by the Psychiatry, Nursing, and Midwifery Research Ethics Subcommittee (PNM RESC), King's College London. REC Reference Number: PNM/14/15-110.

### Recruitment

The survey was advertised on Facebook, Twitter, health and drug related websites and e-mail circulars to include a broad sample of people using codeine resembling the general population. Recruitment lasted between July 2015 and March 2016. The main inclusion criterion was use of codeine containing medicines, prescribed or “over-the-counter,” on at least one occasion in the last 3 months. Only respondents over the age of 18 were asked to participate. Participation in the study was voluntary, anonymous and uncompensated. A more detailed account of the survey has previously been published (26).

### Sample

The survey was embedded within the CODEMISUSED collaboration aiming to carry out national and international studies to estimate levels of codeine use, misuse and dependence in partner countries (Ireland, South Africa and the UK) (27). For this reason, the online survey was open to respondents from all countries. However, in these re-analyses of the data in the present study, it was decided to only include respondents living the UK for several reasons: (i) There is great disparity between levels of codeine consumption, availability of codeine as “over-the-counter” medicines or POM, the amount of codeine included in codeine containing medicines and regulation of advertising of codeine containing medicines across countries around the world (11, 28–30) which may affect aberrant codeine behavior differently. By limiting the sample to the UK, respondents completing the SDS were sourcing and using codeine under similar conditions and regulation; (ii) conducting analysis of the SDS according to nationality was not feasible as some nations were represented by very few respondents; and (iii) the survey was only available in English. Limiting the analysis to respondents living in the UK presumably reduces the risk of misunderstanding due to potential language barriers.

### Procedure

The online survey was developed in Bristol Online Surveys (BOS) and consisted of 49 questions about demographic information, codeine use, codeine dependence, social factors, treatment history, and other substance use (26).

The SDS was included as part of this larger study questionnaire, with the scale items included as questions 28–32 out of a total of 49 questions. The wording of each item of the scale was adapted to enquire about the use of codeine in the last 3 months. Respondents were asked:

- In the last 3 months did you think your use of codeine was out of control? (Responses: “Never/almost never” = 0; “Sometimes” = 1; “Often” = 2; “Always/nearly always” = 3).
- In the last 3 months did the prospect of not taking codeine make you anxious? (Responses: “Never/almost never” = 0; “Sometimes” = 1; “Often” = 2; “Always/nearly always” = 3).
- In the last 3 months did you worry about your use of codeine? (Responses: “Never/almost never” = 0; “Sometimes” = 1; “Often” = 2; “Always/nearly always” = 3).
- In the last 3 months did you wish you could stop taking codeine? (Responses: “Never/almost never” = 0; “Sometimes” = 1; “Often” = 2; “Always/nearly always” = 3).
- In the last 3 months how difficult did you find it to stop using codeine? (Responses: “Not difficult” = 0; “Quite difficult” = 1; “Very difficult” = 2; “Impossible” = 3).

Responses were scored from 0 to 3 for a total score between 0 and 15.

Likely indicators of codeine dependence included frequency of use in the last 3 months, reported as a dichotomous variable (daily or non-daily use). Additional questions were asked about tolerance to codeine and withdrawal symptoms after the use of codeine. Respondents were asked to report if they had sought help to control their use of codeine from (i) a community pharmacist, (ii) a general medical practitioner (GP), or (iii) from the internet. Respondents were also asked about past treatment for alcohol and illicit drug use. The survey included items from the Alcohol, Smoking and Substance Involvement Screening Test (ASSIST) to investigate problems with role responsibility due to the use of codeine (31). Questions from a scale designed to measure reasons for substance use were included to investigate use of codeine for anxiety (32). Several questions about tampering of codeine containing medicines were developed for the study, including about extraction of codeine (otherwise known as “cold water extraction”) (33) and drinking codeine cough syrups mixed with soft drinks or with alcohol. A question about life-time use of illicit drugs, such as cannabis, amphetamines, ecstasy, cocaine, and heroin was included in the survey.

The complete survey was reviewed by experts in codeine misuse and dependence and piloted amongst addiction treatment service users. The survey took between 15 and 20 min to complete.

### Data Analysis

Data were downloaded from the online questionnaires and imported to SPSS. All data analyses were conducted using SPSS, version 24. Before undertaking analyses, all respondents living in countries other than the UK were removed from the dataset. Principal Component Analysis (PCA) was conducted for the five SDS items as proposed by Gossop et al. (14). PCA was applied to determine the number of dimensions and item loading structure. The Cronbach's Alpha coefficient was used to assess the internal consistency of the scale. Mono-variate logistic regression analyses were conducted to estimate the associations between the presence of codeine dependence and individual behaviors relating to codeine use. Comparisons were made between a baseline comparison group consisting of non-dependent codeine users and codeine dependent users. For this part of the analysis, a SDS score of five or above indicated probable psychological dependence on codeine, consistent with previous use of the scale to assess dependence to codeine (25, 34). A score of below five indicated non-dependence. Independent variables were demographic characteristics, frequency of codeine use, tolerance, seeking help to manage codeine, past treatment for alcohol or drug use, social problems, codeine use for emotional distress, tampering of codeine containing medicines, and other substance use.

### Missing Data

To reduce the amount of missing data, most items were mandatory in the computerized survey and respondents could not proceed to the next question without providing an answer. Missing data was therefore uncommon. However, data was missing for amount of codeine consumed on last occasion of use precluding an analysis of this item in the logistic regression model.

## Results

Between July 2015 and March 2016, 472 respondents using codeine in the last 3 months and over the age of 18 completed the survey online. Respondents from outside the UK were removed, leaving a total of 286 respondents in the final analysis. As Table 1 shows, 66.4% of these were female. The mean age of the sample was 35.4 years (SD = 12.5) with a range of 18–71 years. More than half of the respondents were employed full or part-time (60.5%). The main source of obtaining codeine containing medicines was prescribed following a face-to-face consultation with a doctor (45.1% of respondents). The second most common source was purchased “over-the-counter” in a pharmacy without a medical prescription (40.9% of respondents). In the 3 months prior to completing the survey, 39.2% (*n* = 112) of the respondents had consumed codeine daily. A majority of 219 respondents (76.6%) took less or equal to the maximum recommended daily dose of codeine (240 mg), 31 (10.8%) took more and 36 (12.6%) did not provide this information or answered the question incorrectly.

**Table 1:** Respondent characteristics (*n* = 286).

**Characteristic**
Age, mean (SD)	35.4 (SD = 12.5)
**Gender**, ***n*** **(%)^a^**
Female	190 (66.4%)
Male	92 (32.1%)
**Income**, ***n*** **(%)^b^**
Employment	173 (60.5%)
Student allowance	38 (13.3%)
Dependent on other	17 (5.9%)
Disability allowance	17 (5.9%)
No income	10 (3.5%)
Temporary benefit	8 (2.8%)
Pension	7 (2.4%)
Other	8 (2.8%)
**Main type of codeine**, ***n*** **(%)**
Prescription codeine	129 (45.1%)
“Over-the-counter” codeine	117 (40.9%)
Other^c^	40 (14%)

*SD, Standard Deviation*.

a *Four respondents did not say (1.4%)*.

b *Eight respondents did not say (2.8%)*.

c *Purchased online and obtained from friends and family*.

### Variance and Consistency of the SDS

The respondents answered all required questions for the SDS. The responses to the scale produced a full range of scores from 0 to 15 (Mean score = 2.2, SD = 3.5). Principal component analysis (PCA) was undertaken, indicating a single factor solution which accounted for 75% of the variance in codeine dependence (Table 2). The SDS had high internal consistency (Cronbach's alpha = 0.92).

**Table 2:** Factor loadings and percentage of variance accounted for.

	**Respondents (use of codeine in past 3 months) *n* = 286**
SDS score, mean (SD)	2.2 (SD = 3.5)
Range	0–15
**Principal components analysis**	
Number of factors	1
**Factor loadings**	
Item 1	0.89
Item 2	0.88
Item 3	0.87
Item 4	0.87
Item 5	0.83
Item % variance accounted for	75
Cronbach's alpha	0.92

### Associations With Aberrant Codeine Use

Mono-variate logistic regression analyses were used to investigate the relation between codeine dependence and aberrant behaviors in themselves indicating codeine dependence. Non-dependent codeine users were the reference category (Table 3).

**Table 3:** SDS score and its association with aberrant codeine related behaviors.

	**Respondents scoring <5 on the Severity of Dependence Scale (not indicating codeine dependence) (*n* = 235)**	**Respondents scoring ≥5 on the Severity of Dependence Scale (indicating probable codeine dependence) (*n* = 51)**	
	**%**	**%**	**OR (95% CI)**
**Codeine consumption**			
Daily use in last 3 months	26.8%	96.1%^**^	66.89 (15.8–283.18)
**Physical dependence**			
Codeine tolerance	4.3%	58.8%^**^	32.14 (13.82–74.75)
**Sought advice to manage the use of codeine**			
On the Internet	6.8%	41.2%^**^	9.56 (4.5–20.31)
From a GP	2.6%	19.6%^**^	9.31 (3.21–27.01)
**Drug addiction treatment**			
Received treatment to help control alcohol or drug use	12.8%	35.3%^**^	3.73 (1.88–7.43)
**Impact on social life**			
Problems with role responsibility due to codeine	10.2%	52.9%^**^	9.89 (4.95–19.78)
Others expressed concern about use of codeine	10.6%	51%^**^	8.74 (4.39–17.38)
**Emotional distress**			
Used codeine to feel better when down or depressed	13.2%	45.1%^**^	5.41 (2.77–10.55)
Used codeine to stop worrying about a problem	7.7%	33.3%^**^	6.03 (2.83–12.83)
**Codeine tampering**			
Consumed codeine extracted from codeine containing medicines	14.5%	25.5%	1.78 (0.86–3.7)
Drinking codeine cough syrups mixed with soft drink, juice or alcohol	9.4%	25.5%^**^	3.33 (1.55–7.14)
**Illicit drug use**			
Life-time substance use	52.8%	64.7%	1.54 (0.85–2.79)

** *P < 0.01*;

*OR, odds ratio; CI, confidence interval*.

Compared with those who were not dependent on codeine, the group of people with probable codeine dependence were significantly more likely to report daily use of codeine (96.1 vs. 26.8%, *p* < 0.01). In relation to experiences of physical dependence, there was a significantly higher proportion of codeine dependent respondents (58.8%) who reported tolerance to codeine in comparison with non-dependent (4.3%) (*p* < 0.01).

SDS scores were investigated in relation to seeking help to control the use of codeine and specialized addiction treatment history. In the logistic regression model, independent variables that were found to significantly predict probable codeine dependence were having sought help on the Internet (OR = 9.56, 95% CI = 4.5–20.31) and having sought help from a GP (OR = 9.31, 95% CI = 3.21–27.01). Codeine dependent respondents were more likely to have received treatment to manage alcohol and illicit drug use than non-dependent users (35.3% compared to 12.8%, *p* < 0.01).

Those who were dependent on codeine were more likely to report problems with role responsibility, such as missing appointments at work or at home due to intoxication, compared to those who were not dependent (52.9 vs. 10.2%, p < 00.1). SDS scores were investigated in relation to whether a friend or relative or anyone else had expressed concern about the respondents' use of codeine, which was found to significantly predict codeine dependence (OR = 8.74, 95% CI = 4.39–17.38).

Non-medical use of codeine relating to depression and anxiety were found to significantly predict probable codeine dependence, including using codeine to stop worrying about a problem (OR = 6.03, 95% CI = 2.83–12.83) and using codeine to feel better when down or depressed (OR = 5.41, 95% CI = 2.77–10.55).

The group of people with probable codeine dependence had a high proportion of respondents who had consumed codeine cough syrups mixed with soft drinks, juice or alcohol (25.5%) compared to the group of non-dependent respondents reporting this behavior (9.4%) (*p* < 0.01).

There was no significant association between probable codeine dependence and consuming codeine extracted from codeine containing medicines or life-time illicit drug use.

## Discussion

This study demonstrates the feasibility of screening 286 respondents to an online cross-sectional survey for probable codeine dependence using the SDS. Pilot testing of the survey indicated that the five SDS items were easy to understand and the assessment easy to complete. The high questionnaire completion rate to the scale (all items of the scale were completed by all 286 respondents) shows that the SDS was acceptable to use as part of a larger survey study. PCA showed a single factor solution accounting for 75% of the variance. The alpha value was high (Cronbach's alpha = 0.92). Using a score of five and above to indicate probable psychological dependence to codeine, the study demonstrated associations between SDS scores and measures in themselves indicating probable codeine dependence, including daily consumption, tolerance, and problems with role responsibility due to codeine intoxication (25, 35). This compares favorably with a previous study using a similar online research design where probable codeine dependence (indicated by a cut-off score ≥5) was associated with past alcohol and drug addiction treatment, chronic pain, and exceeding medical guidance for dose consumption (25). Online purposive samples have unknown population characteristics (36), but have in this study provided useful preliminary data and indication of using the SDS to assess probable codeine dependence.

### PCA and Consistency of the Scale

PCA has been used to investigate the dimensionality of the SDS for heroin, cocaine, amphetamine and cannabis (14, 37). In this study, PCA indicated a single factor solution accounting for 75% of the variance, suggesting that the five SDS items are suitable as a single measure of psychological dependence. Previous research on the SDS, comparable to findings presented here, found single factor solutions accounting for a range of 45.5–80% of the variance (14, 17, 37).

Cronbach's alpha was used as a measure of internal consistency. According to previous research, values of ≥0.70 were considered adequate (38). An alpha value of 0.92 in the study is equal to or slightly higher than in previous investigations of the scale (14, 24, 37). In addition to the PCA analyses, a high alpha value is also a necessary condition for unidimensionality (14).

While the conducted analyses, including the PCA, satisfy a number of criteria to account for the SDS as a single measure of psychological dependence on codeine, they do not account for how well the SDS determines if respondents have the condition or not. As such the diagnostic properties of the SDS are unclear until further analyses can be completed comparing SDS scores against indicators of codeine dependence from the DSM-5.

### External Validation

Using a cut-off score of five or above, the validity of the SDS score is supported by the association with codeine related behaviors known to be related to the severity of codeine dependence. These include exceeding dose recommendations, daily use, chronic pain, psychological distress, past alcohol and drug addiction treatment, and codeine use to prevent withdrawal symptoms (25, 34, 35). The results obtained in this study show that probable codeine dependence was associated with daily use over the past 3 months, having sought advice and treatment to manage dependence, drinking codeine cough syrup mixed with juice and alcohol, having experienced that other expressed concern about codeine use and using codeine for emotional distress.

A well-known limitation of the SDS is that it was designed to measure psychological elements of dependence, such as compulsion or craving, whilst excluding components relating to physical dependence like tolerance and withdrawal caused by neuroadaptation (14). It is notable in this respect that respondents who were codeine dependent according to the SDS were significantly more likely to report tolerance to codeine than those who were not codeine dependent, supporting the validity of the SDS by its association with this central component of physical dependence.

### Limitations

Whilst, to our knowledge, this is the first study to report on the psychometric properties of the SDS for codeine, the sample size restricts inference of these results to wider populations of people who are using codeine. The sample size is relatively small when considering the time during which the survey was open for recruitment. Lack of data and understanding of codeine dependent populations in the UK impede the construction of a sampling frame and make the representativeness of our sample difficult to measure. Furthermore, it must be noted that online purposive sampling has biases due to unknown characteristics of people who participate in online communities and forums (36). Using online recruitment potentially excludes those with no immediate access to the Internet and may restrict respondents to those with a certain income, social situation and level of education. The differences between levels of codeine dependence and associated problems in online and non-online populations are currently unclear. Missing data precluded an analysis of codeine dose consumption amongst non-dependent and dependent respondents, although dose is a well-known indicator of problematic medicine use (25). Though our findings suggest that a score of 5 and above is an acceptable indicator of probable codeine dependence, the SDS was not designed as a screening tool to decide categorically between non-dependence and dependence (14). Further research is therefore required to compare the adopted cut-off score of 5 against a validated screening tool diagnosing substance dependence. Further research should also explore the use of the SDS compared against a validated diagnostic assessment in different age groups and according to gender.

### Implications for Research

Further studies are needed in well-defined populations to test the different aspects of psychometry of the SDS for codeine to determine its feasibility and validity in research settings. Studies should also investigate the validity of the SDS within different settings, such as primary care, community pharmacies and specialized addiction services. The test-retest reliability of the SDS for codeine is not known. Data that provides an indication of the stability of SDS scores across occasions (39) would add additional value to the scale.

### Implications in Practice

Previous studies have determined a cut-off point on the SDS that discriminates between the presence and absence of a DSM-5 diagnosis for substance dependence suggesting its implementation and usefulness in clinical settings. These studies found a SDS score of 3 or above optimal for characterizing a DSM-5 diagnosis of alcohol dependence (23), whereas a cut-off score of 7 was found to be the appropriate threshold for dependence to benzodiazepines (22). In this study, several factors relating to aberrant codeine use were associated with probable codeine dependence when using a cut-off score of 5. Research with people attending specialized drug addiction treatment for codeine would enable a comparison between SDS scores and DSM-5 diagnosis, possibly enabling its use in clinical settings as a quick way of determining possible psychological dependence on codeine. Obtaining good assessment amongst people presenting with substance use typically improves care and use of screening, assessment and monitoring tools is recommended (40). This study demonstrated that the SDS is useful as a screening tool for research purposes, which can be included in larger study questionnaires with an excellent response rate presumably due to its short length.

## Data Availability Statement

The raw data supporting the conclusions of this article will be made available by the authors, without undue reservation.

## Ethics Statement

The study involving human participants was reviewed, approved and received ethics approval granted by the Psychiatry, Nursing, and Midwifery Research Ethics Subcommittee (PNM RESC), King's College London. REC Reference Number: PNM/14/15-110. The patients/participants provided their written informed consent online to participate in this study.

## Author Contributions

AK, MF, and PD developed the survey used with people who had recently used codeine. The ongoing monitoring of the survey and recruitment was managed by AK. Data analysis was conducted by AK, MF, and JD. All authors contributed to the writing of the paper, with writing and analyses led by PD and AK.

## Conflict of Interest

AK was appointed to the list of experts for 2020–2023 used by the EU drug agency, the EMCDDA, for the assessment of risks posed by NPS. The remaining authors declare that the research was conducted in the absence of any commercial or financial relationships that could be construed as a potential conflict of interest.
